# Multiplex Detection and Genotyping of Point Mutations Involved in Charcot-Marie-Tooth Disease Using a Hairpin Microarray-Based Assay

**DOI:** 10.1155/2009/960560

**Published:** 2009-06-11

**Authors:** Yasser Baaj, Corinne Magdelaine, Virginie Ubertelli, Christophe Valat, Yoanne Mousseau, Hao Qiu, Benoît Funalot, Jean-Michel Vallat, Franck G. Sturtz

**Affiliations:** ^1^Department of Biochemistry and Molecular Genetics, University of Limoges, 87025 Limoges, France; ^2^Serial Genetics, 5 rue Henri Desbruères, 91000 Evry, France; ^3^Department of Neurology, CHU Dupuytren, 87042 Limoges, France

## Abstract

We previously developed a highly specific method for detecting SNPs with a microarray-based system using stem-loop probes. In this paper we demonstrate that coupling a multiplexing procedure with our microarray method is possible for the simultaneous detection and genotyping of four point mutations, in three different genes, involved in Charcot-Marie-Tooth disease. DNA from healthy individuals and patients was amplified, labeled with Cy3 by multiplex PCR; and hybridized to microarrays. Spot signal intensities were 18 to 74 times greater for perfect matches than for mismatched target sequences differing by a single nucleotide (discrimination ratio) for “homozygous” DNA from healthy individuals. “Heterozygous” mutant DNA samples gave signal intensity ratios close to 1 at the positions of the mutations as expected. Genotyping by this method was therefore reliable. This system now combines the principle of highly specific genotyping based on stem-loop structure probes with the advantages of multiplex analysis.

## 1. Introduction

The need for efficient large-scale genotyping methods has rapidly increased over the last ten years. Microarray-based methods offer a convenient solution to this need, but multiplexing is essential to increase the power of this approach for detection and discrimination. This multiplexing can be achieved in several ways, including reducing the complexity of the genome before genotyping. This is achieved by cleaving genomic DNA into fragments with a restriction enzyme and then introducing common adaptor sequences into the restriction products by ligation. These common sequences are then used as binding sites for common PCR primers [1, 2]. This approach is used in high-density microarrays based on the Affymetrix resequencing system (Affymetrix, Santa Clara, Calif, USA) [2, 3]. Long-range PCR for amplifying long PCR fragments, up to 10 kb in length, in each individual PCR has also been described [4, 5]. The Molecular Inversion Probe assay (Parallele Biosciences, South San Francisco, Calif, USA) [6] uses probes that can be circularized and contain two sequences complementary to regions adjacent to SNPs in the DNA target in the first step of the genotyping reaction. These reactions produce templates that can be amplified by PCR, using universal primer sequences. The binding sites for these primers are introduced via the “circularizable” detector probes. The GoldenGate assay (Illumina, San Diego, Calif, USA) [7, 8] is based on a similar principle but includes two oligonucleotides flanking the SNPs for the genotyping reactions. Finally, the pooling of DNA samples or multiplex PCR strategies for SNP genotyping have also been described in several microarray methods, such as the original arrayed primer extension (APEX) [9–12] or single base extension (SBE-tags) [13, 14] assays. 

We previously developed a highly specific genotyping method based on hairpin probes. Our probes, also called hairloops, consist of a GC-rich stem and a target-specific loop [15]. After hybridization with perfectly complementary labeled targets, the stems of probes are opened for the subsequent detection of these targets. One of the major limitations of our original method was the use of individual PCR products, amplified separately, for the hybridization and genotyping reaction. We show here that the multiplex genotyping of multiple mutation sites is possible, based on multiplex PCR DNA amplification and a stem-loop probe method.

## 2. Materials and Methods

### 2.1. DNA Extraction and Gene Mutations

DNA was extracted from blood samples with the Nucleon extraction and purification kit (RPN8512, Amersham Biosciences, GE Healthcare AB, Uppsala, Sweden), used according to the manufacturer's instructions. DNA concentration and purity were determined by spectrophotometry (Shimadzu, UV-160A). All DNA sequences for the three genes known to be involved in CMT disease, for healthy individuals and patients, were confirmed by direct sequencing. The DNA of each patient bores one heterozygous mutation in one of these genes. Four heterozygous point mutations were typed in the three genes implicated in CMT disease. These mutations were the V95M mutation in exon 2 of the GJB1 (Cx32) gene (GenBank accession number XM_047682), the V113F and T124M mutations in exon 3 of the MPZ (P0) gene (GenBank accession number D10537), and the C42R mutation in exon 3 of the PMP22 gene (GenBank accession number L03203). Gene sequences were obtained from GenBank (http://www.ncbi.nlm.nih.gov/). 

### 2.2. Primers and Multiplex PCR Amplification

Three primer pairs were designed for the multiplex assay, generating three amplicons of different sizes. These primer pairs were designed with primer3 software (http://primer3.sourceforge.net/). The sequences of all forward and reverse primers, their selected targets, and the predicted sizes of the PCR products are shown in Table 1. For each pair, the downstream primer corresponded to the 5^′^ end labeled with Cy3. Primers were purchased from Sigma-Proligo (St Louis, Mo, USA) and were 23 to 25 bases long. The primers were designed to amplify the genomic region surrounding the point mutations studied. Part of the long exon 2 of the GJP1 gene, exon 3 of the MPZ (P0) gene, and exon 3 of the PMP22 gene was amplified simultaneously in the multiplex reaction. For PCR, we used the QIAGEN multiplex PCR kit (Qiagen, Hilden, Germany) with 2x QIAGEN multiplex PCR master mix. In a total volume of 50 *μ*L (final concentration, 1x master mix), the concentration of each primer was 0.2 *μ*M and that of MgCl_2_ was 3 mM. We used 100 ng of DNA for each reaction. Amplifications were carried out in a GeneAmp-PCR System 9700 thermal cycler (Applied Biosystems, Foster City, Calif, USA), according to the following protocol: 15 minutes at 95°C (initial activation step), followed by 35 cycles of 94°C for 30  seconds, 68°C for 90 seconds, and 72°C for 90  seconds. The final extension step at 72°C lasted 10 minutes. Amplification products were concentrated and purified with a QIAquick Gel Extraction Kit (Qiagen, Hilden, Germany), and their concentrations were then determined by spectrophotometry (Shimadzu, UV-160A). Finally, 6 *μ*g of each multiplex amplification mixture was hybridized separately to a microarray. 

### 2.3. Microarray, Probes and Hybridization

Microarrays were purchased from Serial Genetics (Evry, France) (http://www.serialgenetics.com/). The probes were designed such that their loops were complementary to the related downstream labeled amplified PCR products. The sequences of wild-type and mutant probes (4 of each) are given in Table 2. Two hairloop probes were designed for each mutation—one for the normal allele and one for the mutant allele—giving a total of eight specific probes. The hybridization mixture consisted of a total volume of 15 *μ*L containing 6 *μ*g labeled multiplex PCR products in 50% formamide, 0.09 M sodium citrate, 0.9 M NaCl, pH 7.1. Before hybridization, this mixture was denatured by heating at 98°C for 3 minutes. Each array was hybridized under a 22 × 22 mm coverslip (Sigma-Aldrich, The Woodlands, Tex, USA) for two hours at room temperature. After hybridization, slides were washed at room temperature for 4 minutes in 0.015 M sodium citrate, 0.15 M NaCl, pH 7.1 (1 × SSC) supplemented with 0.2% SDS, for 3 minutes in 1 × SSC with gentle shaking and then for 3 minutes in 0.1 × SSC with gentle shaking. The slides were dried by centrifugation for 4 minutes at 800 rpm (Jouan CR312, Saint-Herblain, France). 

### 2.4. Scanning and Signal Analysis

The mean Signal-Background (S-B) intensity for each spot was calculated for the five replicates of each spot, using an IMSTAR OSA (*Optical Scan Array)* Reader. S-B intensities were then used for the calculation of discrimination ratios. The DR for each probe was defined as the ratio between the signal obtained on hybridization with the perfect complementary target (i.e., WT target with WT probe, or MT target with MT probe) and the signal obtained on hybridization with the mismatched target (i.e., MT target with WT probe, or WT target with MT probe). The discrimination ratio for the WT target was thus S-B spot WT/S-B spot MT; whereas the discrimination ratio for the MT target was S-B spot MT/S-B spot WT. 

## 3. Results

Genomic DNA from healthy individuals and patients with CMT was separately subjected to multiplex PCR amplification. Cy3-labeled multiplex PCR products derived from each amplification reaction was hybridized and analyzed on a single microarray. After scanning, each sample was tested for the presence or absence of the mutant allele. Discrimination ratios were then calculated to assess the specificity of the method. For a given multiplex reaction hybridization, each experiment performed gave four different DRs: one for each of the mutation positions studied. Each DR indicated the presence of one of two possible genotypes: homozygous WT, resulting from the hybridization of WT targets with WT probes or heterozygous MT, due to the hybridization of MT and WT targets together, each with its perfect complementary probe (i.e., WT target with WT probe, or MT target with MT probe). In our study, each DNA sample with mutated sequences contained only one heterozygous mutation.

Hybridization of the amplified wild-type sequence DNA showed a clear wild-type homozygous genotype, with specific hybridization to WT probes for the four mutation positions, giving strong positive signals. The four calculated DRs for these probes were high and ranged from 17.58 to 73.7. The hybridization of amplified mutant sequence DNA (heterozygous) was also highly specific and gave strong positive signals. These samples were also genotyped correctly. Four DRs were obtained: one corresponding to the existing mutation position and three corresponding to the normal sequence for the other three mutation positions studied. The calculated DRs of these hybridizations at the existing mutation position ranged from 0.24 to 3.2 and were systematically below the value of five arbitrarily defined as the threshold for the genotyping of heterozygous sequences. These DRs gave heterozygous MT genotypes. The DRs obtained for the other three positions (each WT) in this hybridization indicated homozygous WT genotypes and were above 14 (data not shown). Only DRs at the positions of the mutations after the hybridization of DNA from patients were considered from the four DRs obtained for calculation of the power of discrimination between genotypes. 

**Figure 1:**
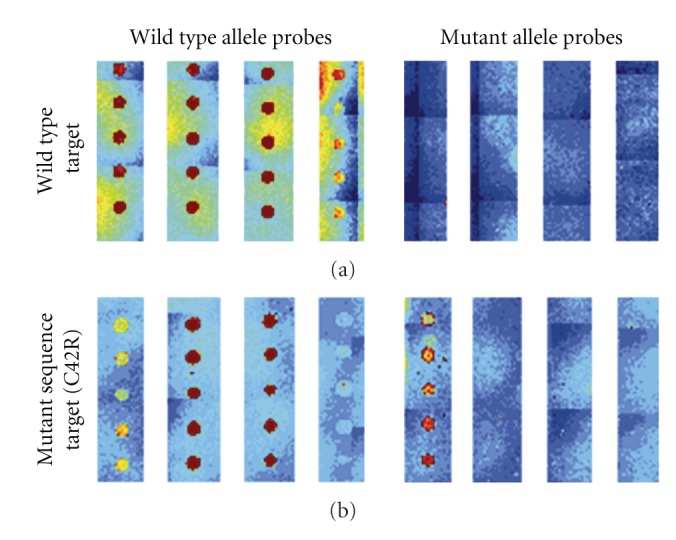
*Hybridization specificity*. Each spot type on the microarray was presented in five vertical replicates. (a) Hybridization of amplified DNA from healthy individuals (WT). All WT probe types corresponding to hybridized targets were detected. (b) Hybridization of mutant sequence DNA (C42R). WT and MT probes for the heterozygous C42R mutation were clearly detected with a discrimination ratio of 2.2. All other WT spots corresponding to hybridized targets were also detected.

Power of discrimination between genotypes was also calculated by dividing the ratio of signals from normal and mutant alleles at a homozygous position (DR, homozygous) after WT sequence hybridization with the ratio of signals at a heterozygous position (DR, heterozygous) after heterozygous MT sequence hybridization. This discrimination power was high, ranging from 19 to 211 for each mutation position.  Table 3 provides a summary of the discrimination ratios and discrimination powers obtained in this study, and Figure 1 provides an example of the typical results obtained following multiplex amplified DNA hybridization for a healthy individual or a patient (C42R MT). 

## 4. Discussion

Microarray approaches linked to nucleic acid analysis are emerging as powerful tools for the multiplex genotyping of SNPs and large-scale mutation screening. In this study, we established proof-of-principle for the use of hairpin-shaped probe microarrays for genotyping multiple mutation positions and rapidly analyzing DNA sequences by hybridization. Each pair of probes (WT and MT) was previously tested in individual PCRs and was demonstrated to five correct genotypes [15]. In this study, we set up a fluorescent multiplex polymerase chain reaction (PCR) for amplifying four loci located in three different genes in the same reaction. DNA from healthy individuals and from patients with CMT disease was analyzed on hairloop arrays designed to detect the presence or absence of heterozygous mutations. The four loci in the multiplex system provide useful information about polymorphism for mutation genotyping. The multiplexing procedure used did not affect specificity but did increase efficiency. The signal intensities of wild-type probes were 18 to 74 times higher than those of the homologous mutant sequence probes differing by a single nucleotide (DR) from healthy individual “homozygous” DNA. “Heterozygous” mutant DNA samples gave signal intensity ratios close to 1 at mutation positions, as expected for wild-type and mutant sequence signals of approximately equivalent intensity. 

The reported values for (S-B) of wild-type allele probe (S-B) of mutant allele probe, and consequently discrimination ratios were different between our herein study and our previous publication [15]. This difference is mainly of technical nature and is due to the fact that the hybridization signals and ratio values strongly depend on the specific and proper background value of each array. This background value is somehow correlated to the quantity of hybridized DNA and fluctuates between arrays even when using the same washing conditions. For these reasons, ratios of (S-B) values corresponding to wild type or mutant alleles were used for calculating discrimination ratios. 

After multiplex PCR product hybridization, the discrimination ratio for a given mutation concerning wild type “homozygous" DNA samples was lower than that obtained with individually amplified samples [15]. This difference may be due to the reduced availability of hairpin probes for hybridization due to the high quantity (6 *μ*g) of multiplex PCR products used here. Moreover, the noncomplementary PCR products to a given probe could, by their quantity, prevent hybridization of other PCR products with their corresponding complementary probes. The raised background value, which generates a decrease in S-B values after hybridization of 6 *μ*g multiplex PCR products, is an additional cause for this difference of DRs. What is very important to note though, is that S-B values of the two alleles for “heterozygous" mutant DNA samples were always almost equal and conserved a DR close to 1.

The high quantity of hybridized DNA should be then reconsidered in the future. The amount of multiplex PCR product used for hybridization could be decreased by increasing labeling efficiency. This could be achieved by incorporating several labeled nucleotides during amplification or by labeling the DNA after PCR amplification, using commercial kits specially designed for this purpose. Methods used for the amplification of single stranded PCR products or for breaking the target into smaller fragments would also be required in this approach. Our intention here was to demonstrate that it is feasible to multiplex hairloop-based microarrays. We think that the ability to genotype SNPs with multiplex PCR reactions and easily generated specific microarrays would make this approach effective, rapid, and inexpensive.

Finally, we compared our system with other known microarray-based genotyping systems for multiplex SNP detection. Some of these methods, such as SBE-tags [13] and APEX [11, 16, 17] methods, have levels of multiplexing and genome complexity reduction similar to those of our method. However, the use of different labeled dideoxynucleotides by these methods increases the cost. Moreover, these methods require the inclusion of an extension step, in addition to clean-up and heat inactivation steps, none of which are necessary in our method, which is therefore simpler and faster.

Other multiplexing methods such as long-range PCR [4, 5] or the use of universal primers [1, 2], based on the use of allele-specific hybridization on high-density microarrays (e.g., Affymetrix “GeneChip" arrays); have also been described. These assays use large numbers of probes (10 to 14) per SNP variant and require very large microarrays [18], whereas our method is based on the use of a single probe for each variable nucleotide. These microarrays must be also produced by specialized industrial companies. The signals obtained with these arrays are generally acquired and analyzed with special scanners and software obtained from the manufacturer and developed especially for these purposes. This greatly increases the cost of these systems, which are not cost-effective for genotyping intermediate numbers of SNPs, as in our system. 

The Molecular Inversion Probe (Parallele Biosciences) [6] and GoldenGate (Illumina) [7, 8] assays are also subject to this problem. Both require several long specialized multiplexing procedures. Specific sets of probes or oligonucleotides bearing particular tag sequences must be designed for each SNP site. This increases the complexity of this assay, resulting in larger setup costs.

Our method is clearly simpler and faster than most of these tests and could be presented as a more cost-effective and time-saving alternative particularly for intermediate (tens to hundreds of sites) numbers of SNPs ($50/sample). It would be straightforward to adapt this method to individual academic research laboratories with classical scanners and array equipment. The short hybridization step (2 hours) at room temperature is also an important feature of our method, in addition to the low costs of primer synthesis, due to a single 5^′^ amino linker modification. The hairloop method, with its corresponding multiplexing power, may be modified for various applications and may hold considerable promise, not only for the multiplex detection of SNPs, but also for gene profiling and expression studies.

## Figures and Tables

**Table 1:** Genes, detected mutations and primers used for multiplex PCR and amplified fragment lengths.

Primers	Mutation	Gene	Exon amplified	*T *m	GC%	Fragment length (bp)	Primer sequence
Multi 1 (F)	V95M	Cx32	Exon 2	67.20	45.83	574 bp	AGCTTGCTTCATGGCTGGTGTTTT
Multi 1 (R)				67.15	59.09		ACTGTGTTGGGGCAGGGGTAGA
Multi 2 (F)	V113F	P0	Exon 3	67.86	52.00	285 bp	CTCTCACATGCTTCCCCTCATTCCT
Multi 2 (R)	T124M			67.70	52.00		CAAACTGCTTCCCATACCCTTGTCC
Multi 3 (F)	C42R	PMP22	Exon 3	67.78	52.00	197 bp	TCCTTCCCCTTTTCCTTCACTCCTC
Multi 3 (R)				67.66	48.00		ACAAGCTCATGGAGCACAAAACCAG

**Table 2:** Probe sequences.

Gene mutation	WT spotted probe sequence	MT spotted probe sequence
V95M	GCGCCGATGCACGTGGCTCACGGCGC	GCGCCGATGCACATGGCTCACGGCGC
V113F	GCGAGCGCTCCATTGTCATACACAAGCTCGC	GCGAGCGCTCCATTTTCATACACAAGCTCGC
T124M	GCGAGCCAATGGCACGTTCACTTGCTCGC	GCGAGCCAATGGCATGTTCACTTGCTCGC
C42R	GCGAGCCAGAACTGTAGCACCGCTCGC	GCGAGCCAGAACCGTAGCACCGCTCGC

**Table 3:** Genotyping, discrimination ratios, and discrimination powers for healthy individuals and patients with CMT.

Mutation	Sample genotype	(S-B) of wild-type allele probe	(SB) of mutant allele probe	Discrimination ratio	DNA type	Power of discrimination between genotypes
V95M	Homozygous Wild-type	33.4	1.9	WT Probe: 17.58	Normal control	19
	Heterozygous	8.6	8	MT Probe: 0.93	Patient	
V113F	Homozygous Wild-type	233.65	3.17	WT Probe: 73.7	Normal control	23
	Heterozygous	111.8	362.7	MT Probe: 3.2	Patient	
T124M	Homozygous Wild-type	181.09	3.58	WT Probe: 50,58	Normal control	211
	Heterozygous	138.3	33.9	MT Probe: 0.24	Patient	
C42R	Homozygous Wild-type	141.18	2.15	WT Probe: 65.66	Normal control	30
	Heterozygous	55.6	126	MT Probe: 2.2	Patient	

Signal-Background (S-B) calculations for each spot bearing the wild-type or mutant allele were obtained after the hybridization of PCR products from heterozygous patients and unaffected individuals. The discrimination ratio between mutant and normal allele probes should be as high as possible for healthy individuals (homozygous wild-type genotypes) and as close as possible to 1 for heterozygous affected patients. The discrimination power should be as high as possible.

## Abbreviations

CMT: Charcot-Marie-Tooth disease
CHR: Cross-hybridization rate
DR: Discrimination ratio
WT: Wild-type sequence of probe or target
MT: Mutant type sequence of probe or target, for example,
V95M WT: Probe or target bearing the wild-type allele of the V95M mutation;
V95M MT: Probe or target bearing the mutant allele of the V95M mutation.
